# Comparison of unidimensional and bidimensional measurements in metastatic non-small cell lung cancer

**DOI:** 10.1038/sj.bjc.6600449

**Published:** 2002-07-02

**Authors:** J Cortes, J Rodriguez, J A Diaz-Gonzalez, C Garzon, A Gurpide, L Arbea, I Gil-Bazo, V Navarro, M Cambeiro, A I Nicolas, S Martin-Algarra, J Garcia-Foncillas, E Calvo

**Affiliations:** Department of Oncology, Clínica Universitaria de Navarra, Avenida de Pío XII, 36, 31008 Pamplona, Spain; Department of Radiology, Clínica Universitaria de Navarra, Avenida de Pío XII, 36, 31008 Pamplona, Spain; Department of Oncology, Gregorio Marañón Hospital, 28007 Madrid, Spain; Department of Oncology, San Jaime Hospital, 03280 Torrevieja, Alicante, Spain

**Keywords:** RECIST, WHO, response, non-small cell lung carcinoma, chemotherapy

## Abstract

Tumour response evaluation after chemotherapy has become crucial in the development of many drugs. In contrast to the standard bidimensional WHO criteria, the recently described Response Evaluation Criteria In Solid Tumors are based on unidimensional measurements. The aim of the present study was to compare both methods in patients with metastatic non-small cell lung cancer. One hundred and sixty-four patients treated with two cisplatin-paclitaxel-based chemotherapy schedules between June 1994 and December 2000 were analysed. The measurements were reviewed by an independent panel of radiologists. Patient characteristics were: median age of 55 years (range 24–77 years) and a male to female ratio of 129 : 35. Adenocarcinoma and squamous carcinoma were the most common histologies. Vinorelbine was the third drug used in 77 patients and gemcitabine in 87. The ratio unidimensional/bidimensional was as follows: response 85 : 85; stable disease 32 : 32; progression 47 : 42 and not assessable 0 : 5. Kappa for agreement between responders was 0.951 (95% CI: 0.795–1.0) (*P*<0.001). Both WHO criteria and Response Evaluation Criteria In Solid Tumors give similar results in assessing tumour response in patients with non-small cell lung cancer after chemotherapy. The unidimensional measurement could replace the more complex bidimensional one.

*British Journal of Cancer* (2002) **87**, 158–160. doi:10.1038/sj.bjc.6600449 www.bjcancer.com

© 2002 Cancer Research UK

## 

During the last decades there has been a growing interest in defining, exactly and uniformly, objective tumour shrinkage after treatment with different anticancer agents. It was necessary to find a common language to allow investigators to compare their results in an appropriate way. This issue was first elucidated when the World Health Organization (WHO) (WHO handbook, 1979) published its tumour objective response criteria to antineoplasic therapy, based on bidimensionally measurable lesions (report summarised by Miller *et al*, 1981). Different response categories were identified (complete response, partial response, stable disease and progression) and defined as an arbitrary percentage. To establish the response category, it was necessary to compare the product of the maximum and perpendicular diameters of all measurable lesions before and after therapy. However, some practical problems were encountered: in addition to being very laborious, the methods for assessment of the lesions and the number of these varied among the different investigation groups, a situation that could in itself introduce variability into the outcomes of the different groups.

Many efforts have been made to improve the currently available criteria. James *et al* (1999) suggested that the information obtained from single dimensional measures could be equivalent to the bidimensional ones when determining the tumour response to treatment. They developed a theoretical basis for this, as follows: a one-dimensional measurement of tumour lesions better defines the proportion of tumour cells killed by a certain dose of an antineoplasic agent rather than a bidimensional product. In this way, they established a mathematical relationship between the criteria of the WHO (diameter product) and their own proposition using a single measure. Based on this model, new guidelines for evaluating the response to treatment in solid tumours (RECIST) were put forward (Therasse, 2000).

Hypothetically, unidimensional measurement of tumour lesions may substitute for the usual bidimensional one. The aim of the present study was to determine whether the unidimensional (RECIST) approach can be compared with the bidimensional (WHO) criteria in non-small cell lung cancer.

## METHODS

To evaluate the hypothesis by which unidimensional and bidimensional measurements should produce similar response rates in patients with non-small cell lung cancer, we analysed objective tumour responses in 164 patients treated with triple-drug paclitaxel and cisplatin-based chemotherapy combinations between June 1994 and December 2000. Treatment was administered every 4 weeks. To be entered into this retrospective study, patients had to fulfil all of the following criteria: a histologically or cytologically proven stage IV NSCLC with at least one bidimensionally measurable lesion greater than or equal to 1×1 cm in size, taking into account that the minimum size of the lesion should be no less than double the slice thickness, no previous systemic therapy and ECOG performance status of 0–2. No restrictions due to extent of disease were imposed.

Pre-treatment evaluation consisted of a complete medical history and physical examination, complete blood cell (CBC) count with white blood cell differential and platelet counts, standard biochemical profiles, chest and abdomen computer tomographic (CT) scans, magnetic resonance imaging (MRI) of the brain, and bone scan. On-treatment evaluation included physical examination, monitoring of toxic effects and evaluation of measurable lesions with CT scans and/or MRI at the beginning of each cycle. Intravenous contrast agents to accentuate vascular structures and oral contrast agents to highlight the bowel against other soft-tissue masses were consistently administered, and all images were available for review at both soft tissue and lung settings. Lesions were then measured on the same window setting on each examination.

Each patient's tumour measurements were evaluated for response according to both WHO criteria and RECIST. Complete response was defined as the disappearance of all evidence of tumour, as well as signs, symptoms and biochemical changes related to the tumour for at least 4 weeks, during which no new lesions should appear (WHO criteria), and as the disappearance of all target and nontarget lesions and the normalisation of tumour marker level confirmed by repeat assessments that should be performed no less than 4 weeks after the criteria for response is first met, during which no new lesions should appear (RECIST evaluation). Partial response was defined as: (1) WHO – a 50% or greater reduction in the sum of the products of the two largest perpendicular diameters of all measurable lesions that persisted for at least 4 weeks; or (2) RECIST – a greater than or equal to 30% decrease in the sum of the largest unidimensional measurements, maintained for a minimum of 4 weeks. Stable disease was defined for both WHO and RECIST as change in the sum of the products or diameters, respectively, insufficient for partial response and progressive disease, maintained for a minimum of 4 weeks from baseline. The WHO criterion for progression is a >25% increase in the sum of the product of the two largest perpendicular diameters of one measurable lesion (even with regression of the remaining lesions), or the appearance of any new lesion, and the RECIST is a >20% increase in the sum of the largest unidimensional measurements or the appearance of one or more new lesions.

To be assigned a status of partial response or complete response, changes in tumour measurements must be confirmed by repeat assessments that should be performed no less than 4 weeks after the criteria for response are first met and overall response categorisation of patients depends on the classification of tumour response in both target and non-target lesions with or without the appearance of new lesions according to RECIST.

Three radiologists performed the imaging assessments, working independently. Target lesions were selected on the basis of their size and their suitability for accurate repeat measurements according to the reviewer criteria. In case of disagreements, they were resolved by consensus.

The primary goal of this study was to compare the response rates by WHO criteria and RECIST. Evaluation of stable and progressive disease by both methods was also done. To estimate their concordance, the k statistic was used (Landis and Koch, 1977). It would be positive if the agreement was more than would be expected by chance, and would be unity if there was a total concordance between both criteria.

## RESULTS

A total of 164 chemotherapy-naive patients with metastatic NSCLC treated with a paclitaxel-cisplatin based therapy between June 1994 and December 2000 were analysed. The median age was 55 years (range 24–77 years). The majority of patients had good performance status. The male to female ratio was 129 : 35. Adenocarcinoma and squamous histologies were found in 84 and 61 patients, respectively. Seventy-seven patients received vinorelbine as third agent and cisplatin-paclitaxel with gemcitabine were administered to 87 patients.

The measurements were reviewed by an independent panel of radiologists. Overall responses according to WHO criteria and RECIST are summarised in Table 1

**Table 1:**
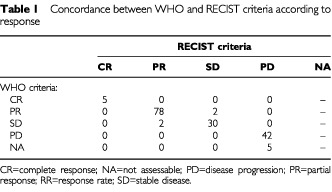
Concordance between WHO and RECIST criteria according to response

. There were five complete responses by each of both measurement criteria. The concordance regarding partial response was excellent, with 80 responders to each category of criteria. Only two patients (1.2%) were considered to be responders by WHO but not by RECIST, and other two were judged to be responders by RECIST, but were not considered to be responders by WHO. Thus, there was an agreement in 78 of 80 responders. Stable disease was observed in 32 patients by each of the two measurements. Five patients could not be evaluated for response by WHO criteria, four of them due to early toxic death and the other one because of pulmonary embolism 4 weeks after the therapy was initiated. All of them were considered, by definition, a ‘best response’ of progression according to the RECIST. Thus, 47 patients and 42 patients showed progressive disease by the unidimensional and the bidimensional measurements, respectively.

Overall response is shown in Table 1. Concordance between both methods of measurement was excellent. Kappa for agreement between RECIST and WHO responses was 0.951 (95% CI: 0.795–1.0) (*P*<0.001).

## DISCUSSION

Although WHO criteria are considered to be a standard method for evaluation of treatment results, a unidimensional measurement-based set of response evaluation criteria in solid tumours has been recently proposed as a valid alternative. The aim of the present study in patients with NSCLC was to determine the degree of agreement between tumour response after treatment measured by the classical WHO criteria and measured by the new RECIST approach.

As might be expected, the agreement between WHO and RECIST regarding complete response was total. The concordance in partial response was excellent too; there was an agreement in 78 out of 80 partial responders, so almost the same patients were considered to be responders by either method. However, 47 patients showed progressive disease by the unidimensional method and 42 patients by the bidimensional one. Either early death from malignant disease or early death from toxicity, or early death from another cause are considered to be a failure to respond to treatment by RECIST, whereas these cases were considered to be ‘not assessable for response’ by WHO. This has been one of the reasons why different results from the same therapy have been given in different reports. In our study, 42 patients progressed really, and the remaining five patients died early. Therefore, it should be taken into account that all patients who enter into a study are ‘evaluable’ for response by RECIST with the advantage that an incorrect treatment schedule or drug administration does not result in exclusion from the analysis of the response rate. Although the volume relationship between the WHO criteria for progressive disease (25% sum product increase; 40% volume size) and RECIST (20% sum diameter increase; 73% volume increase) was not the same, in our study there was a complete concordance in all the patients who progressed due to an increase in measurement of pre-existing lesions. This was probably due to the great number of patients who progressed because of the appearance of new lesions.

To our knowledge, there have been few studies in the literature assessing the agreement between the two criteria in NSCLC. James *et al* (1999) reported results comparing 24 patients with NSCLC treated with paclitaxel and ifosfamide. Interestingly, there was complete concordance between responders, but four patients showed progressive disease and 16 stable disease by WHO, with only one patient progressing and 19 showing stable disease by the unidimensional criteria. A greater than or equal to 30% increase in the sum of the largest unidimensional measurements or the appearance on any new lesion was necessary to define the disease as ‘progression’, a fact that could have had a negative influence on the agreement between both methods according to progressive disease. Although the RECIST guidelines were based on the model proposed by James *et al* (1999), they consider a 20% sum diameter increase to be enough for progression. In a second study, Watanabe *et al* (2000) reported results after evaluating 99 patients and concluded that unidimensional measurement may be sufficient for evaluating the tumour response to chemotherapy for NSCLC, but these data have not yet been published in a peer-reviewed medical journal. In their study, 18 patients showed partial response by each method. Although Grossi *et al* (2001) reported consistency in measurements by WHO and RECIST, these data should be viewed with caution because the sample size was very low (three patients). In another report, Werner-Wasik *et al* (2001) in a well-designed study, investigated whether tumour largest dimension, bidimensional tumour product, and volume correlate with each other in evaluating locally advanced NSCLC. They concluded that any of the three tumour measurements could be used as a reliable tool in assessing lung cancer response. Only 22 patients were analysed, but the overall response rate was identical (86%) by both unidimensional and bidimensional criteria.

In conclusion, the high degree of concordance for overall response rate judged by the WHO criteria and RECIST suggests that both methods are equally useful. Moreover, the summing of diameters is easier, faster and is an ongoing indicator of how tumour burden is changing. Therefore, unidimensional measurement may be sufficient for the evaluation of tumour response to therapies for NSCLC.
